# Method of Ascertaining before Accouchement Whether the Mother Will Have Sufficient Milk

**Published:** 1845-05

**Authors:** 


*Method of Ascertaining before Accouchement whether the Mother will have sufficient Milk.*—" Donné brings forward the following propositions with the greatest confidence in their truth, and after having tested them by a very considerable experience.

" Pregnant women, considered in the relation of the secretion of colostrum, during the last months of gestation, may be distributed into three classes :—

" 1st. Those in whom this secretion is, so to say, absent, and from whom it is impossible to obtain, by a regulated pressure, more than a drop, or half a drop of a liquid presenting to the microscope some few milky globules, with granular bodies swimming in a troubled or viscid liquid.

" 2d. Those in whom the secretion is more abundant, and from whom there can be drawn with facility a fourth or half-glass of colostrum, offering the following characters to the microscope: milky globules few, and of a middle size, or numerous and very small; these globules, sometimes badly formed, swim in a liquid of little density; they are mixed with a certain number of granular bodies, and sometimes we find at the same time mucous globules.

" 3d. The third class comprehends those women in whom the secretion of the mammary gland is not only abundant, but in whom it is also rich in globules of a good size, being, for the most part, 1-100th to 1-30th of a millimetre, in diameter; they are well-formed, and nearly as regular as in perfect milk; it presents, likewise, the oily drops, granular bodies, &c., of the colostrum.

"Now, if the first kind of colostrum exist before birth, you may be sure that the milk subsequently will be serous, poor, and insufficient for the nourishment of the child. In the second category, also, the milk, after accouchement, may be very abundant, but it will be poor and serous. The third kind of colostrum, however, indicates always a milk equally rich and abundant.—*Dublin Journal* in *Bulletin of Medical Science.*

